# Bioinformatic and mass spectrometry identification of *Anaplasma phagocytophilum* proteins translocated into host cell nuclei

**DOI:** 10.3389/fmicb.2015.00055

**Published:** 2015-02-06

**Authors:** Sara H. G. Sinclair, Jose C. Garcia-Garcia, J. Stephen Dumler

**Affiliations:** ^1^Graduate Program in Cellular and Molecular Medicine, The Johns Hopkins University School of MedicineBaltimore, MD, USA; ^2^Department of Pathology, The Johns Hopkins University School of MedicineBaltimore, MD, USA; ^3^Department of Pathology, University of Maryland School of MedicineBaltimore, MD, USA; ^4^Department of Microbiology and Immunology, University of Maryland School of MedicineBaltimore, MD, USA; ^5^Procter and Gamble Co.Cincinnati, OH, USA

**Keywords:** *Anaplasma phagocytophilum*, nucleomodulin, nuclear translocation, oxidative burst, iTRAQ

## Abstract

Obligate intracellular bacteria have an arsenal of proteins that alter host cells to establish and maintain a hospitable environment for replication. *Anaplasma phagocytophilum* secrets Ankyrin A (AnkA), via a type IV secretion system, which translocates to the nucleus of its host cell, human neutrophils. *A. phagocytophilum*-infected neutrophils have dramatically altered phenotypes in part explained by AnkA-induced transcriptional alterations. However, it is unlikely that AnkA is the sole effector to account for infection-induced transcriptional changes. We developed a simple method combining bioinformatics and iTRAQ protein profiling to identify potential bacterial-derived nuclear-translocated proteins that could impact transcriptional programming in host cells. This approach identified 50 *A. phagocytophilum* candidate genes or proteins. The encoding genes were cloned to create GFP fusion protein-expressing clones that were transfected into HEK-293T cells. We confirmed nuclear translocation of six proteins: APH_0062, RplE, Hup, APH_0382, APH_0385, and APH_0455. Of the six, APH_0455 was identified as a type IV secretion substrate and is now under investigation as a potential nucleomodulin. Additionally, application of this approach to other intracellular bacteria such as *Mycobacterium tuberculosis, Chlamydia trachomatis* and other intracellular bacteria identified multiple candidate genes to be investigated.

## Introduction

*Anaplasma phagocytophilum* is an obligate intracellular bacterium of human neutrophils. The neutrophil is an unlikely host as it creates an intracellular milieu that is a highly inhospitable environment for bacterial survival. Yet, *A. phagocytophilum* requires the neutrophil for propagation and survives by altering the cellular antimicrobial properties while paradoxically increasing pro-inflammatory functions (Banerjee et al., 2000; Carlyon et al., 2002; Borjesson et al., 2005; Choi et al., 2005; Carlyon and Fikrig, 2006). The fitness advantage gained with suppression of microbial killing while enhancing recruitment of new host cells for population expansion is the benefit of this paradoxical dichotomy of functional reprogramming. There is increasing evidence to suggest that the bacterium accomplishes this with coordinated reprogramming of neutrophil gene transcription by reorganizing large regions of host cell chromatin (Sinclair et al., 2014).

Importantly, *A. phagocytophilum* produces a protein, Ankyrin A (AnkA) that is exported from the bacterium and eventually localizes to the nucleus of the infected host cell (Caturegli et al., 2000; Park et al., 2004). Previously, our laboratory investigated the effect of infection on the transcriptional repression of *CYBB*, encoding gp91*^phox^* (Garcia-Garcia et al., 2009a,b). AnkA is capable of directly binding host cell DNA, and in the case of *CYBB*, transcription is dampened when AnkA binds to its proximal promoter (Park et al., 2004; Garcia-Garcia et al., 2009a,b). Furthermore, increased histone deacetylase (HDAC) activity enhances *A. phagocytophilum* infection in part because AnkA recruits HDAC1 to the *CYBB* promoter to close the chromatin and exclude RNA polymerase binding (Garcia-Garcia et al., 2009a; Rennoll-Bankert and Dumler, 2012). Owing to their capacity to enter the nucleus and modulate host cell transcription, microbial factors such as AnkA have been called “nucleomodulins.”

It is currently unclear as to whether HDAC recruitment is the predominant mechanism by which AnkA exerts its chromatin modulating effects, whether there are other host factors (e.g., polycomb repressive or hematopoietic associated factor-1 [HAF1] complexes), or additional bacterial-derived nucleomodulins that further contribute to reprogramming. The *A. phagocytophilum* genome encodes a type 4 secretion system (T4SS) that allows the bacteria to translocate effector proteins into the host cytosol (Dunning Hotopp et al., 2006; Lin et al., 2007; Rikihisa et al., 2010). AnkA was the first T4SS substrate identified among Rickettsiales, and it plays a critical and potentially dominant role in the course of establishing and sustaining neutrophil infection (IJdo et al., 2007; Garcia-Garcia et al., 2009a,b; Al-Khedery et al., 2012; Rennoll-Bankert and Dumler, 2012). In contrast to other gram-negative T4SSs, the *vir* genes encoding the secretion system of the *Rickettsiales* family are organized differently in that they are clustered in three different genomic locations (Ohashi et al., 2002; Rikihisa et al., 2010). Between individual *A. phagocytophilum* strains, variations of the T4SS appear to contribute to host specificity and strain virulence (Al-Khedery et al., 2012).

We hypothesize that *A. phagocytophilum* expresses additional nuclear effector proteins secreted by its type IV secretion system (T4SS) and that these also play a role in pathogenicity. It is likely that some will be nucleomodulins which could contribute to transcriptional reprogramming of infected neutrophils. Pilot studies using bioinformatics tools, and iTRAQ protein profiling among infected and uninfected cells were used to identify candidate proteins that potentially localize to the host cell nucleus. The profiling identified 50 *A. phagocytophilum* proteins, one of which was AnkA, and at least 7 of these were predicted to enter the nucleus based on the presence of both a nuclear localization sequence and a bacterial secretion signal sequence. Ultimately, 3 of the 7 proteins identified in the bioinformatic screen and 3 of 37 identified by iTRAQ profiling of nuclei from infected cells translocated into HEK-293T human embryonic kidney and PLB-985 granulocytic cell nuclei.

## Methods

### *In silico* prediction of *A. phagocytophilum* proteins targeted to the host cell nucleus

Our initial focus was on proteins involved in regulation of host gene expression. Since these events occur chiefly in the nucleus, we developed an unbiased computational approach to identify potential nucleomodulins encoded in intracellular bacterial genomes based on their likelihood for translocation into the host cell nucleus and applied this to the *A. phagocytophilum* HZ strain genome (Supplemental Figure 1). Annotated protein tables for bacteria, focusing on the *A. phagocytophilum* HZ strain genome, were obtained from the National Center for Biotechnology Information (NCBI) database (ftp://ftp.ncbi.nih.gov/genomes/Bacteria). The *A. phagocytophilum* protein table was used as the database for eukaryotic subcellular localization search algorithms. Although we used a database with 1264 annotated *A. phagocytophilum* proteins, including hypothetical proteins, multiple programs were implemented to obtain high prediction accuracy and processing capacity. Since we needed only to predict nuclear proteins, localization coverage was not taken into account. Since hybrid methods are preferable when little is known about the protein of interest (Donnes and Hoglund, 2004) we used ProtComp Version 6 (Softberry, Inc.), a computational algorithm for the identification of sub-cellular localization of eukaryotic proteins.

We next applied PSORTb v.2.0 to exclude potential membrane proteins that are unlikely to be secreted into the host cell (Gardy et al., 2005). Finally, we used computational algorithms to predict the presence of eukaryotic nuclear localization signals (NLS). NLSs often possess sequences with a high basic amino-acid content (Hicks and Raikhel, 1995) and are generally classified into three categories: classical or monopartite (NLSm), bipartite (NLSb), and a type of N-terminal signal found in yeast protein, Mat alpha2, a poorly studied signal that is not incorporated in most NLS prediction algorithms. To screen broadly for potential NLSs, we selected MultiLoc (Hoglund et al., 2006). MultiLoc also identifies matches in NLSdb, a database of experimentally known NLSs (Nair et al., 2003) and is also useful to predict NLSm and NLSb in addition to the NLSdb attribute, since NLSdb recognizes only 43% of the nuclear proteins. MultiLoc calculates a probability estimate for each subcellular location and the protein is assigned to the compartment with the highest score. The MultiLoc output was recorded and used to calculate a Nuclear Score that better reflects the purpose of the search:

$$\begin{array}{l} \text{Nuclear Score = MultiLoc Nuc + NLSdb }\\ \;+(0.5\times \text{NLSm}+0.5\times \text{NLSb}) \end{array}$$

where: (i) 0 ≤ MultiLoc Nuc <1 is the probability estimate of the protein being nuclear as calculated by MultiLoc; (ii) NLSdb is 1 if the protein contains a known NLS, 0 if not; (iii) NLSm is 1 if the protein contains a predicted NLSm, 0 if not; and (iv) NLSb is 1 if the protein contains a predicted NLSb, 0 if not. Weighting was applied since the presence of a predicted NLS suggests, but is not conclusive; therefore the NLSm or NLSb prediction contributes only half to the final nuclear score. The addition of the continuous MultiLoc Nuc score provides a better ranking of the proteins, given that the other indicators contribute discretely to the Nuclear Score. However, no proteins without a known or predicted NLS will produce a Nuclear Score >1 since MultiLoc Nuc <1.

### *iTRAQ* for identification of potential nuclear-translocated protein profiling

*A. phagocytophilum*-infected and uninfected HL-60 cells, a promyelocytic cell line commonly used for *A. phagocytophilum* propagation as previously described (Goodman et al., 1996; Park et al., 2004), were fractionated to obtain nuclei and nuclear proteins. iTRAQ (isobaric tag for relative and absolute quantitation protein profiling technology [Applied Biosystems]), a mass spectrometric technique where 2 protein expression profiles are compared, was used to identify candidate bacterial proteins present in the nucleus of infected cells. One hundred μg in replicate samples from nuclear fractions of infected and uninfected HL-60 cells were acetone-precipitated and checked for protein integrity and sample quality. The samples were reduced and cysteines blocked following the iTRAQ kit protocol (Applied Biosystems). Samples were digested with trypsin overnight at 37°C and then labeled with iTRAQ tags in replicates, pooled and fractionated using a strong cation exchange (SCX) column on an Ultimate HPLC system (LC Packings). Approximately 20 fractions were collected and analyzed on Qstar Pulsar™ (Applied Biosystems-MDS Sciex) interfaced with an Agilent 1100 HPLC system. Peptides were separated on a reverse-phase column, and MS/MS analysis was performed. The MS/MS spectral data were extracted and searched against Uniprot-sprot database (entries for *Homo sapiens* and *A. phagocytophilum*) using ProteinPilot™ software (Applied Biosystems). For each protein, two types of scores were reported: unused ProtScore and total ProtScore. The total ProtScore is a measurement of all the peptide evidence for a protein and is analogous to protein scores reported by other protein identification software programs. However, the unused ProtScore is a measurement of all the peptide evidence for a protein that is not better explained by a higher ranking protein and was the method of choice. The protein confidence threshold cutoff for this study was set at an unused score of 2.0 with at least one peptide with 99% confidence. A ratio of infected to uninfected (Aph:HL-60) score was used to identify *A. phagocytophilum* proteins in nuclear lysates. To do this, we averaged the ratios of uninfected HL-60 nuclear lysate replicates (isobaric isotope labels 115:114) and ratios of nuclear lysate replicates from *A. phagocytophilum*-infected HL-60 cells (116:114 and 117:114) to create the composite Aph:HL-60 mean ratio. Proteins identified with mean ratios (infected/uninfected) >1.2 were selected for further study.

### GFP-fusion protein plasmid clones and transfections

Forty one GFP C-terminal fusion proteins were prepared using pMAXFP-Green-C (Lonza, cat# AMA-VDF1011) and the Infusion HD Liquid cloning kit (Clontech). Briefly, target genes were amplified using PlatinumTaq (Life Technologies) and PCR purified using Qiagen PCR purification kits (Qiagen). Primers were designed using Clontech's Online Infusion tools, Primer Design. Amplicons were created to be fused with the pMAXFP-Green-C vector after digestion with *Xho*I. Primers were approximately 40–45 bp in length and had the sequence GAAGAAAGATCTCGAGCT added to the 5′ end of the forward gene-specific primer (20–25 bp), and GAAGCTTGAGCTCGAGT added 5′ to the reverse primer (Supplemental Table 1). The Infusion-HD kit instructions were followed as per the manufacturer's recommendations. Clones were transformed into *E. coli* JM109 (Promega) and after antibiotic selection, were sequenced to ensure they were in the correct orientation and in frame. HEK-293T cells were transfected with GFP-fusion vectors using Lipofectamine 2000 (Life Technologies) and PLB-985 cells were transfected with the Amaxa Nucleofector shuttle and the SF kit reagents (Lonza) as per manufacturer's recommendations. PLB-985 cells are human myelomonoblast leukemia cells that easily differentiate into neutrophil-like cells and are readily transfected as opposed to HL-60 cells, a common host cell model for *A. phagocytophilum* infection (Pedruzzi et al., 2002; Ellison et al., 2012; Rennoll-Bankert et al., 2014). Cells were stained with DAPI 24 h later and imaged by fluorescence microscopy, gathering both superimposed green fluorescent protein and DAPI images.

### Determination of T4SS substrates

*A. phagocytophilum* proteins that localized to the nucleus of HEK-293T and PLB-985 cells were tested for their ability to be secreted by the T4SS Dot/Icm system of *Coxiella burnetii* RSA439 avirulent phase II nine-mile strain using adenylate cyclase translocation assays (Larson et al., 2013). Fusion proteins were created by cloning full-length coding regions or C-terminal 100 aa truncations to the *Bordetella pertussis* adenylate cyclase gene (*cyaA*). To achieve this, *C. burnetii* was transiently propagated in ACCM-2 axenic culture medium and transformed with the constructs (performed at the NIAID Rocky Mountain Laboratories [Hamilton, MT] by Paul Beare, Ph.D. and Charles L. Larson). Axenic *C. burnetii* was transformed by electroporation and cultured in ACCM-2 medium for 24 h followed by chloramphenicol selection (Beare et al., 2009; Voth et al., 2011). The ability of the constructs to be secreted by the Dot/Icm system was determined by measuring changes in intracellular cAMP levels. CyaA fusion proteins that contain a T4SS signal are capable of being secreted and mediate a measurable increase in cAMP. *C. burnetii* transformants containing the *cyaA* constructs were used to infect THP-1 cells (a human myelomonocytic cell line) at an MOI of 100:1, and included both *A. phagocytophilum* AnkA (APH_0740) and *Coxiella* vacuolar protein A (CvpA), both known T4SS substrates as positive controls. After 3 days, the cells were harvested, lysed and examined for cAMP production by enzyme immunoassay. Results were expressed as fold change in intracellular cAMP concentration compared to empty vector control (CyaA only) that lacked a T4SS signal; values >2 were considered positive for type 4 secretion; values between 1 and 2 were considered marginal.

### Assay for detection of reactive oxygen species

Superoxide production was detected as described previously (Rennoll-Bankert et al., 2014). Briefly, HL-60 cells were incubated with 0.25 mM 2′,7′-dichlorofluoresein diacetate (DCFH-DA) in PBS for 30 min at room temperature. 10^5^ cells were stimulated in triplicate with 1 μg/mL phorbol 12-myristate 12-acetate (PMA) and fluorescence was measured every 2 min. The relative fluorescence units at 180 min were averaged and compared to unstimulated controls using a two-sided Student's *t*-test, α 0.05.

## Results

### *In silico* prediction of *A. phagocytophilum* proteins targeted to the host cell nucleus

Of 1264 proteins and hypothetical proteins examined by the bioinformatics algorithm, 123 were identified by ProtComp as nuclear-localized; 3 of these were classified in PSORTb as potentially nuclear membrane-associated; after analysis of NLSdb and screening for NLSm and NLSb, 7 candidate proteins had a total Nuclear score >1 (Table 1). One candidate with a high ProtComp score for nuclear localization but that lacked a predicted NLS (APH_0805) was selected as a control. The known nuclear-translocated AnkA was not identified in this screen.

**Table 1:** **Bioinformatic prediction of *A. phagocytophilum* nuclear-translocated proteins, by likelihood based on Final Score rank**.

**Locus name**	**Acc. No**.	**Protein**	**Gene**	**Ranking**	**ProtComp**	**MultiLoc**	**NLS**	**NLS1**	**NLS2**	**Final score**
APH_0820^a^	YP_505397.1	Hypothetical protein		10	2.1	0.94	1	1	0	2.44
APH_0847	YP_505424.1	Hypothetical protein		22	2.1	0.97	0	1	1	1.97
APH_0382	YP_504988.1	HGE-14 protein		56	1.7	0.97	0	1	0	1.47
APH_0385	YP_504990.1	HGE-14 protein		75	2.1	0.94	0	1	0	1.44
APH_0455	YP_505057.1	HGE-14 protein		76	2.2	0.94	0	1	0	1.44
APH_0485	YP_505084.1	Hypothetical protein		77	2.2	0.94	0	1	0	1.44
APH_0576	YP_505167.1	RNA polymerase sigma factor RpoD	*rpoD*	114	2.1	0.89	0	1	0	1.39
APH_0805^b^	YP_505382.1	Hypothetical protein		1891	2.1	0.96	0	0	0	0.96

a *Not cloned*.

b *Selected as negative control*.

### *iTRAQ* identification of *A. phagocytophilum* nuclear-translocated proteins

We detected 43 *A. phagocytophilum* proteins with an Aph:HL-60 ratio >1.2 in the nucleus of infected cells (Table 2), including the top hit, AnkA that is established to translocate into the nucleus. This approach allowed the identification of *A. phagocytophilum* proteins most likely to have been translocated into the nucleus and provided a more complete list of candidates to investigate out of the 1264 *A. phagocytophilum* ORFs available for study. Of these 43 candidates, only AnkA was excluded from subsequent cloning and expression for *in vitro* nuclear localization studies.

**Table 2:** ***Anaplasma phagocytophilum* proteins identified in the nuclear lysates of infected HL-60 cells by iTRAQ with ratios compared with uninfected cells of >1.2 and ranked by Unused ProtScore to identify high likelihood candidates for nuclear translocation**.

**Locus name**	**Accession**	**Protein**	**Gene**	**Ratios of labeled peptides^a^**			**Mean HL-60**	**Mean Aph**	**Ratio Aph:HL-60**	**Unused ProtScore**
				**115:114**	**116:114**	**117:114**				
APH_0740	gi|88607707	Ankyrin A	*ankA*	1.06	1.42	1.43	1.03	1.43	1.38	40.10
APH_1023	gi|88607105	DNA-directed RNA polymerase subunit beta; RNAP subunit beta	*rpoC*	1.04	1.50	1.39	1.02	1.45	1.42	32.10
APH_0240	gi|88606723	60 kDa chaperonin GroEL	*groEL*	1.00	1.88	1.93	1.00	1.90	1.90	30.60
APH_1024^2^	gi|88606872	DNA-directed RNA polymerase subunit beta; RNAP subunit beta	*rpoB*	1.02	1.33	1.27	1.01	1.30	1.28	23.40
APH_0906	gi|88606911	Hypothetical protein APH_0906		1.05	1.25	1.22	1.02	1.24	1.21	20.70
APH_0278^2^	gi|88607578	Translation elongation factor Tu; EF-Tu	*tuf1*	1.18	1.93	1.83	1.09	1.88	1.73	20.20
APH_1099	gi|88607685	DNA-binding response regulator CtrA	*ctrA*	1.05	2.65	2.71	1.03	2.68	2.62	19.90
APH_0303	gi|88606699	DNA-directed RNA polymerase subunit alpha; RNAP subunit alpha	*rpoA*	1.16	1.91	1.84	1.08	1.88	1.74	15.90
APH_0784	gi|88606926	DNA-binding protein HU	*hup*	1.00	2.38	2.03	1.00	2.21	2.21	15.40
APH_0968	gi|88606840	ATP-dependent protease La	*lon*	1.01	1.62	1.44	1.01	1.53	1.52	15.10
APH_1100	gi|88606714	DNA-binding protein		0.99	2.82	2.44	0.99	2.63	2.64	13.80
APH_0469	gi|88607025	Putative malonyl-CoA decarboxylase		1.04	1.27	1.28	1.02	1.27	1.24	12.60
APH_0445	gi|88607683	Transcription elongation factor NusA	*nusA*	1.00	1.53	1.50	1.00	1.52	1.52	12.20
APH_0339	gi|88607311	Putative thermostable metallocarboxypeptidase		1.11	1.59	1.58	1.05	1.58	1.50	9.60
APH_1239	gi|88607921	P44–15b outer membrane protein; major surface protein-2C	*p44–15b*	1.05	3.60	3.63	1.03	3.62	3.53	9.10
APH_0062	gi|88606901	Hypothetical protein APH_0062		1.06	1.91	1.77	1.03	1.84	1.79	8.70
APH_1097	gi|88607712	DNA polymerase III, beta subunit	*dnaN*	1.14	1.33	1.30	1.07	1.32	1.23	6.60
APH_0135	gi|88606701	Cold shock protein, CSD family		0.97	1.79	1.78	0.99	1.79	1.81	6.30
APH_0397	gi|88606909	30S ribosomal protein S2	*rpsB*	1.07	1.53	1.45	1.04	1.49	1.44	6.20
APH_1263	gi|88607227	Translation initiation factor IF-3	*infC*	0.94	2.12	1.97	0.97	2.05	2.11	5.00
APH_0398	gi|88607503	Elongation factor Ts; EF-Ts	*tsf*	1.03	1.24	1.28	1.01	1.26	1.24	4.40
APH_1151	gi|88607101	Hypothetical protein APH_1151		1.20	2.16	2.11	1.10	2.13	1.94	4.10
APH_0288	gi|88607038	50S ribosomal protein L29	*rpmC*	1.03	1.77	1.66	1.01	1.72	1.69	4.10
APH_1029	gi|88607731	Transcription termination/antitermination factor NusG	*nusG*	0.94	1.65	1.72	0.97	1.69	1.74	4.00
APH_1027	gi|88607420	50S ribosomal protein L1	*rplA*	1.15	1.37	1.26	1.08	1.31	1.22	3.30
APH_0515	gi|88606905	Expression regulator ApxR	*apxR*	0.99	2.02	1.94	0.99	1.98	2.00	3.20
APH_0097	gi|88606982	Protein-export protein SecB	*secB*	1.11	1.81	1.67	1.06	1.74	1.64	3.00
APH_0292	gi|88606711	50S ribosomal protein L5	*rplE*	1.26	1.52	1.57	1.13	1.54	1.36	2.40
APH_0106	gi|88607568	Riboflavin synthase, alpha subunit	*ribE*	0.98	2.04	2.00	0.99	2.02	2.04	2.30
APH_0280	gi|88607449	50S ribosomal protein L3	*rplC*	1.07	1.60	1.64	1.04	1.62	1.56	2.30
APH_0629	gi|88607793	Malate dehydrogenase	*mdh*	0.98	1.25	1.21	0.99	1.23	1.24	2.30
APH_0160^2^	gi|88606875	Putative thymidylate synthase, flavin-dependent, truncation		1.14	1.50	1.37	1.07	1.44	1.34	2.20
APH_0154	gi|88607134	Serine hydroxymethyltransferase SHMT	*glyA*	1.06	1.29	1.26	1.03	1.27	1.24	2.10
APH_0971	gi|88607838	Trigger factor; TF	*tig*	1.04	1.37	1.28	1.02	1.32	1.30	2.00
APH_0659	gi|88607183	Antioxidant, AhpC/Tsa family		0.99	1.26	1.23	1.00	1.24	1.25	2.00
APH_1349	gi|88606948	Glyceraldehyde-3-phosphate dehydrogenase, type I	*gap*	0.86	1.13	1.19	0.93	1.16	1.24	2.00
APH_1198	gi|88606994	2-oxoglutarate dehydrogenase, E2 component, dihydrolipoamide succinyltransferase	*sucB*	0.98	1.24	1.16	0.99	1.20	1.21	2.00
APH_1025	gi|88607605	50S ribosomal protein L7/L12	*rplL*	1.01	1.85	1.79	1.01	1.82	1.81	1.90
APH_0289^2^	gi|88607574	30S ribosomal protein S17	*rpsQ*	0.90	1.57	1.46	0.95	1.52	1.60	1.70
APH_1034^2^	gi|88607212	30S ribosomal protein S7	*rpsG*	1.16	1.42	1.41	1.08	1.41	1.31	1.50
APH_0196^2^	gi|88607673	Response Regulator NtrX, putative nitrogen assimilation regulatory protein	*ntrx*	0.93	1.70	1.50	0.97	1.60	1.66	1.40
APH_1333^2^	gi|88607617	Transcription elongation factor GreA	*greA*	0.95	1.29	1.27	0.98	1.28	1.31	1.30
APH_1098	gi|88607131	3′–5′ exonuclease family protein		1.12	1.30	1.29	1.06	1.30	1.22	1.30

a *Isobaric ion labels of nuclear lysates from: 114 and 115, uninfected HL-60 cells; 116 and 117, A. phagocytophilum-infected HL-60 cells*.

^b^ *Not cloned*.

### *In vitro* nuclear localization

**Figure 1:**
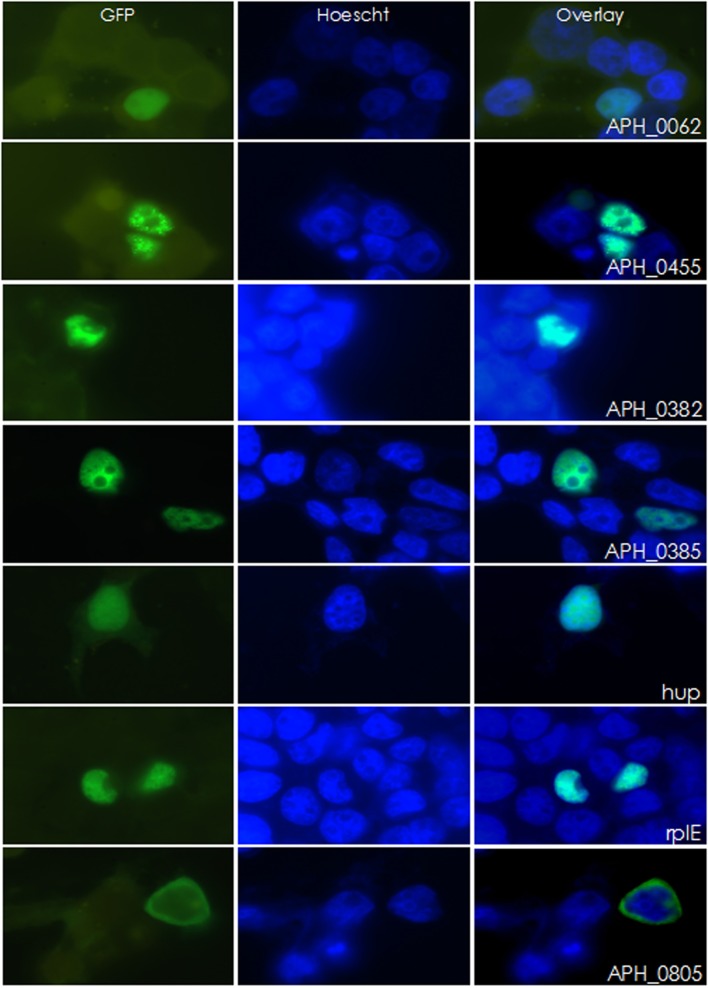
**Six *A. phagocytophilum* candidate genes were found to localize to the nucleus of HEK-293T cells**. Candidate genes were fused to GFP and transfected into HEK-293T cells. Twenty four hours post-transfection, cells were stained with DAPI and imaged. Of the 42 GFP-fusion proteins created, six localized to the nucleus. APH_0805 is shown here as an example of a protein that did not localize to the nucleus.

As an inclusive screen, and because contamination of nuclear preparations could not be entirely excluded in iTRAQ studies, nuclear localization of proteins identified by bioinformatic methods or by iTRAQ mass spectrometry were confirmed by cloning the corresponding genes into a mammalian expression vector for expression as GFP fusion proteins. APH_0805 that was predicted to have nuclear localization yet lacked a predicted NLS and had a below-threshold Nuclear score was used as a non-translocating control. HEK-293T cells and PLB-985, a promyelocytic cell line, were transfected and examined for nuclear localization of the GFP-fusion proteins with Hoescht 33342 nuclear counterstaining. Six of the 42 proteins tested (36 from iTRAQ profiling, 7 from the bioinformatic screen), translocated to the nucleus: APH_0062 (hypothetical protein), RplE (50S ribosomal protein L5 [APH_0292]), Hup (DNA-binding protein HU [APH_0783]), and APH_0455, APH_0382, and APH_0385 (all HGE-14) (Figure 1 and Supplemental Figure 2). APH_0278 (*tuf-1*; elongation factor Tu) was not cloned, but instead the identical APH_1032 (*tuf-2*; elongation factor Tu) was used but did not enter the nucleus. Nine proteins were either unable to be cloned or cloning was not attempted, including: APH_0160 (putative thymidylate synthase, flavin-dependent, truncation, partial); APH_0196 (nitrogen assimilation regulatory protein); APH_0289 (ribosomal protein S17 [*rpsQ*]); APH_0820 (hypothetical protein); APH_0906 (hypothetical protein); APH_1023 (DNA-directed RNA polymerase, beta subunit [*rpoC*]); APH_1024 (DNA-directed RNA polymerase, beta subunit [*rpoB*]); APH_1034 (ribosomal protein S7 [*rpsG*]) and APH_1333 (transcription elongation factor GreA).

### Determination of type 4 secretion substrates

**Figure 2:**
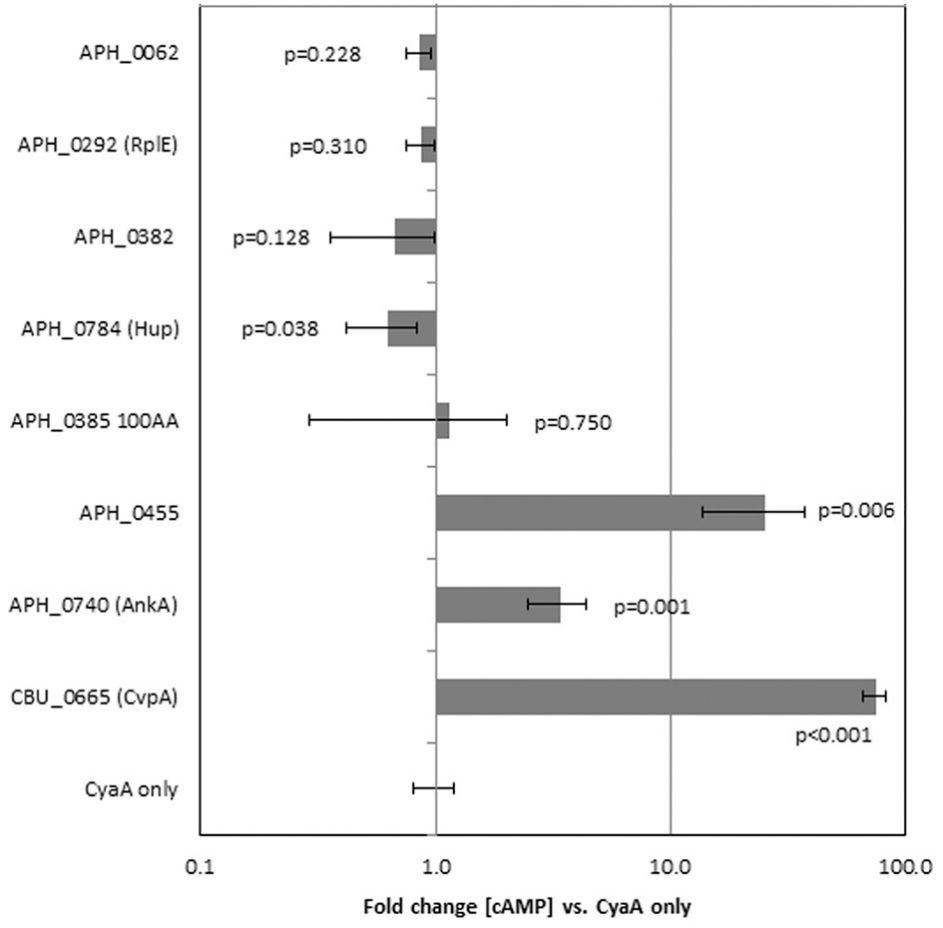
**APH_0455 is secreted by the *Dot/Icm* T4SS of *C. burnetii***. Candidate genes were fused to *B. pertussis* adenylate cyclase (*cyaA*), transfected into *C. burnetii* and selected by chloramphenicol resistance. Transformed *C. burnetii* clones were then used to infect THP-1 cells. Three days post-transfection, THP-1 cells were assayed for cAMP production. Only those constructs that contain a T4SS signal sequence have measurable changes in cAMP production. The results represent the average of two separate experiments each with replicate tests. The *p*-values were calculated based on comparisons with fold change of *C. burnetii* transformed by empty plasmid (CyaA only) using two-sided Student's *t*-tests, α = 0.05. CBU_0655 is CvpA, a known T4SS substrate of *C. burnetii*.

Proteins identified to localize to the nucleus were further investigated to determine if they could be secreted by the T4SS of *Coxiella burnetii*, which is similar to that of *A. phagocytophilum*. T4SS substrate status was determined by the ability of the CyaA-fusion to exit *C. burnetii* and produce a measurable increase in cAMP concentrations with infection of THP-1 cells. Of the 6 genes tested only APH_0455 was identified to be a type 4 secretion substrate (Figure 2).

### Determination of oxidative burst after transfection and nuclear translocation

**Figure 3:**
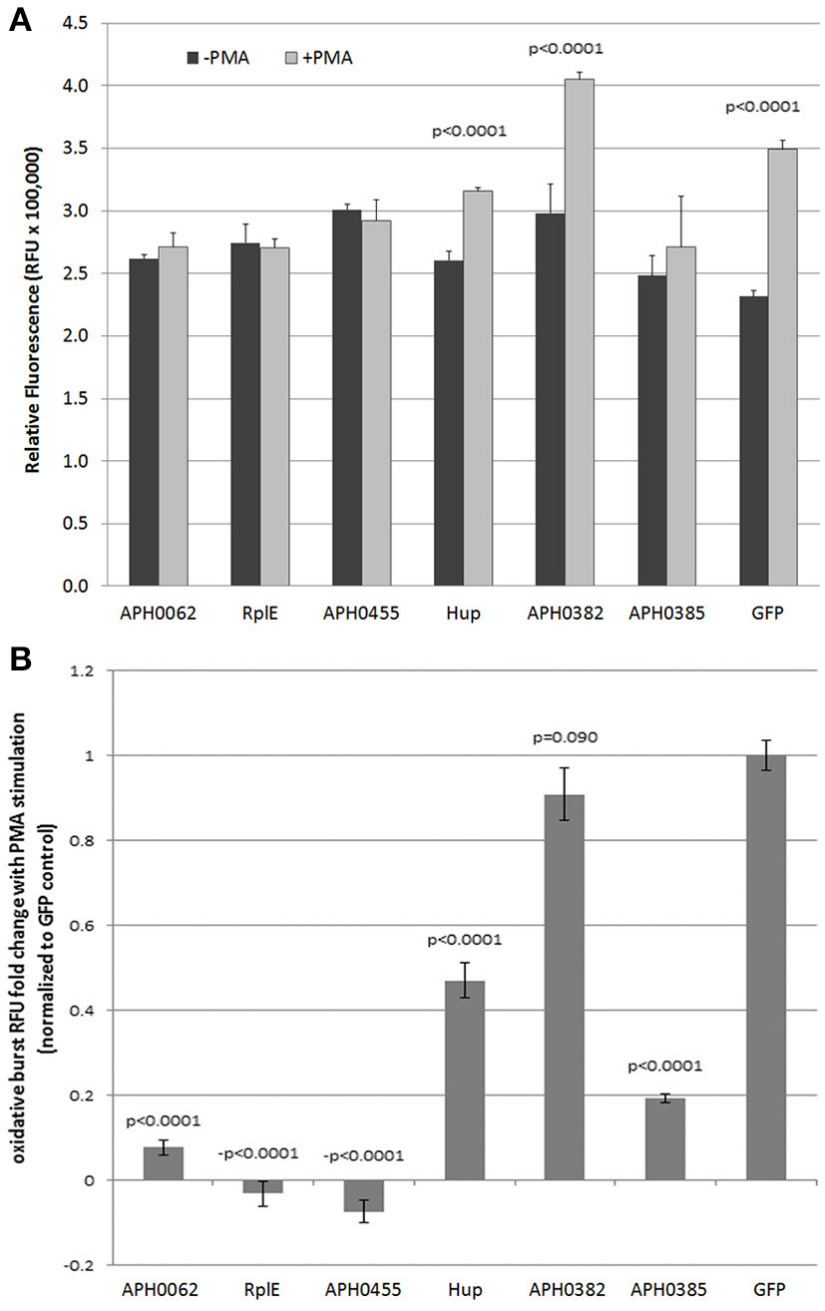
**Expression of putative nuclear effectors APH_0062, RplE and APH_0455 dampen PMA-stimulated reactive oxygen species production by HL-60 cells**. HL-60 cells were transfected with 2 μg plasmid and assayed for respiratory burst 48 h later. **(A)** The average of three replicates is displayed ±SEM at 180 min. **(B)** The fold change was calculated by dividing the ratio of PMA stimulation of each transfectant to the GFP control. *P*-values were calculated using Students *t*-tests.

GFP-fusion constructs were transfected into HL-60 cells to determine their ability to alter the oxidative burst response. Unfortunately, the methods (electroporation, lipofectamine, viral transduction) used to transfect the HL-60 cells (differentiated or undifferentiated), abrogated oxidative burst as compared with non-transfected cells. Thus, we compared results to PMA-stimulated oxidative burst in HL-60 cells transfected with the empty GFP plasmid as control. When RFU values of each unstimulated transfected control cell culture were compared to PMA-stimulated, significant oxidative burst, as seen with the GFP plasmid control, was observed only with Hup and APH_0382 (Figure 3A). When normalized to GFP plasmid transfection alone, APH_0062, RplE, APH_0455, Hup, and APH_0385 significantly repressed respiratory burst (Figure 3B). However, responses varied in intensity over several repeated experiments, likely in part due to the variable transfection efficiency obtained with HL-60 cells. These data suggest that one or more of these effectors could contribute to dampened production of reactive oxygen species.

## Discussion

While considerable focus has been placed on AnkA as the primary nucleomodulin of *A. phagocytophilum*, it does not seem plausible that a single protein can account for the widespread transcriptional and phenotypic changes induced with infection. Using current bioinformatics tools and mass spectrometry, a number of other proteins encoded in the *A. phagocytophilum* genome were identified that could potentially localize to the host cell nucleus. To validate the candidate genes, GFP-fusion proteins were created and screened for nuclear localization within HEK-293T cells. This approach narrowed the list of target genes for further investigation to six.

No candidate proteins were identified in both the bioinformatic screen and in the iTRAQ mass spectrometry analysis. If one assumes that the mass spectrometry data is accurate, the bioinformatic approach was ineffective at identifying features to predict nuclear localization for 3 of the six proteins shown capable of entering the nucleus; as a result, APH_0062 (cytoplasmic), *hup*, and *rplE* (both mitochondrial) were excluded from the bioinformatic identification because they were not assigned a nuclear localization. In contrast, no bioinformatic-predicted candidate appeared in the iTRAQ mass spectrometry analyses, suggesting limitations in sensitivity and/or contamination of nuclear preparations by non-nuclear localized proteins. Thus, the combination of both approaches increased the ability to identify and exclude candidates for further analysis. It is important to note that the screen will only identify those genes capable of entering the nucleus on their own accord via an identified or unidentified nuclear localization signal. Some proteins identified as present in the nucleus in the iTRAQ screen could indeed localize to the nucleus but might not be confirmed by transfection screens. A bacterial-derived protein shuttled into the nucleus as a component of a protein complex, or one that possesses an uncharacterized NLS, as is the case with AnkA, would not be identified. Furthermore, HEK-293T cells are not a model cell line for *A. phagocytophilum* infection and transfection of these proteins does not mimic infection, a much more complex process; therefore, confirmation of nuclear translocation in PLB-985 was performed.

Additionally, *A. phagocytophilum* is largely refractory to gene delivery by genetic transformation. Previous reports demonstrate *A. phagocytophilum* transformation using the Himar1 transposase system that introduces small GFP proteins or disrupts bacterial genes and consequently protein expression (Felsheim et al., 2006; Chen et al., 2012). This process does not result in gene entry, but results in a library of mutant bacteria that can facilitate complex functional studies and insight into the importance of mutated genes for establishing or maintaining infection. However, directed mutation by homologous recombination has not yet been described for *A. phagocytophilum*. None-the-less, this relatively simple experiment yielded multiple candidate genes of interest for further investigation.

After narrowing the initial bioinformatic and iTRAQ list of candidate genes to six, we investigated the ability of these proteins to be secreted by the bacterium. For *A. phagocytophilum*, the most well characterized secretion mechanism is that of the T4SS. Because of this, we focused on whether or not these proteins could be secreted by a T4SS. As an obligate intracellular bacterium that resides solely in membrane-bound vacuoles of its host cells, *A. phagocytophilum*-secreted proteins very likely first enter the cytosol before translocation to the nucleus, but are unlikely to be detected outside of the host cell owing to the intracellular vacuolar membranes accessible to the bacterium. Thus, the *C. burnetii* Dot/Icm T4SS was used as a surrogate delivery system because *C. burnetii* is capable of being transfected easily when cultivated in axenic medium but resides within host cell vacuoles when cultivated in mammalian cells. The *Dot/Icm* secretion system is compatible with that of *A. phagocytophilum* and, unless cultivated in specific axenic medium, *C. burnetii* is also an obligate intracellular bacterium residing within membrane-bound vacuoles. Using fusions with *B. pertussis* CyaA, one of six *A. phagocytophilum* candidate nuclear-localizing proteins was identified as a T4SS substrate. The remaining 5 did not appear to be secreted by the *Dot/Icm* system. Using SignalP 4.1 (http://www.cbs.dtu.dk/services/SignalP/), we determined the presence of putative Sec1 secretion signals in the genes encoding APH_0382, APH_0385, and APH_0455 (all HGE-14-like); experimental confirmation of this secretion mechanism was not further attempted.

Interestingly, APH_0382, APH_0385, and APH_0455 were shown to be differentially expressed between mammalian and tick cells. The transcription of each of these proteins was approximately 2.9–3.3-fold greater in HL-60 cells than ISE6 (tick) cells (Nelson et al., 2008). This suggests that these HGE-14-like proteins likely play a role in establishing or maintaining infection in mammalian cells. In fact, differential transcription of *A. phagocytophilum* genes plays a role in the life cycle of the bacterium in mammalian and tick cells (Wang et al., 2007; Nelson et al., 2008; Troese et al., 2011; Mastronunzio et al., 2012). APH_0784 (DNA binding protein HU), and APH_0292 (50S ribosomal protein L5) are among the 20 most abundant proteins expressed in infected *I. scapularis* salivary glands (Mastronunzio et al., 2012), and both were found in nuclear lysates of infected HL-60 cells, yet predicted to localize to the mitochondrion and cytosol, respectively. We also identified the transcriptional regulator of p44/*msp2* genes, ApxR (APH_0515; Wang et al., 2007) in nuclear lysates from *A. phagocytophilum* infected HL-60 cells, but at a low unused ProtScore. As ApxR was unable to translocate to the nuclei of HEK293 cells, its presence indicates the potential for low level cytoplasmic contamination in the nuclear preparations. However, the overall level of cytoplasmic contamination is likely to be low since the most abundant *A. phagocytophilum* proteins in the P44/Msp2 family (Wang et al., 2007; Nelson et al., 2008; Mastronunzio et al., 2012) were not abundant in nuclear lysates. Finally, APH_1235 is characterized as a specific marker of dense core infectious *A. phagocytophilum* (Troese et al., 2011). It is among the 20 most abundantly-expressed proteins in tick salivary glands (Mastronunzio et al., 2012), is significantly upregulated in dense core cells with HL-60 cell infection (Troese et al., 2011), and is believed to facilitate tick to mammal transmission. While predicted to localize to the nucleus by ProtComp v.6 and identified in infected HL-60 cell nuclear lysates, published data demonstrate the lack of nuclear localization (Troese et al., 2011). Moreover, it lacked a recognized NLS and the iTRAQ unused score was low, suggesting low-level contamination from the host cytosol.

APH_0455, a HGE-14 protein, is of particular interest owing to its utilization of the T4SS to enter the cell and its translocation into the nucleus where it forms small aggregates and clusters dispersed unevenly throughout the nucleoplasm. APH-0455 is one of several HGE-14 proteins predicted to enter the nucleus, and APH-0455 has been described to have transmembrane domains that would predict it to be a type II membrane protein, and possesses 4 conserved 41 amino acid repeats followed by 2 similar truncated repeats (Lodes et al., 2001). This repeat region overlaps a region with a conserved Med15/ARC15 (pfam09606) domain. Med15/ARC105 domains are found as part of a family of sterol regulatory element binding proteins (SREBPs), transcription activators that regulate genes involved in cholesterol and fatty acid homeostasis. In humans, SREBPs bind CREB-binding protein (CBP)/p300 acetyltransferase that in turn affect chromatin structure and gene transcription (Yang et al., 2006). Whether APH_0455 plays a role in these critical pathways for *A. phagocytophilum* survival needs to be determined (Lin and Rikihisa, 2003).

Because of the candidate proteins' abilities to act as T4SS or Sec1 substrates and to localize to the nucleus, we sought to determine if they played a role in altering the phenotype of HL-60 cells, a commonly used cell model for *A. phagocytophilum* infection. Unfortunately, transfection of HL-60 cells with a variety of methods inconsistently altered oxidative burst capacity, and often the vehicle controls and transfection reagents were enough to abrogate responses. Despite the variable responses, we observed trends toward reduction of oxidative burst (Figure 3). Despite these trends, we cannot currently conclude with certainty that these effectors play a role in limiting oxidative burst as shown for AnkA (Banerjee et al., 2000).

For each of the *A. phagocytophilum* proteins that localized to the nucleus of HEK-293T and PLB-985 cells, it would be important confirm their presence in the nuclei of *A. phagocytophilum*- infected cells visually or biochemically, and to potentially assess the effects of their absence in *A. phagocytophilum* among Himar1 transposase libraries (Nelson et al., 2008; Troese et al., 2011). Additionally, future studies will examine their role in transcriptional and functional changes in differentiated HL-60 cells, the preferred model for *A. phagocytophilum*-directed neutrophil reprogramming. Such studies will focus on transcriptional responses, functional assays and, given the role that AnkA plays during the course of infection, studies of nuclear protein-protein, DNA-protein, and RNA-protein interactions. The screening techniques modeled here using *A. phagocytophilum* will allow for a more focused approach to identify potential nucleomodulins and could facilitate studies of microbial nucleomodulin manipulation of host cell transcriptional programs.

These techniques are not limited to the *A. phagocytophilum* genome but can also be applied to other intracellular bacteria. Using the same bioinformatics approaches (Supplemental Methods, Supplemental Figure 1, Supplemental Tables 1, 2), candidate genes were identified for other pathogens including, but not limited to: *Chlamydia trachomatis, Coxiella burnetii, Ehrlichia chaffeensis, Mycobacterium tuberculosis, Yersinia pestis, Legionella pneumophila, Francisella tularensis*, and *Listeria monocytogenes*. Identification of new nucleomodulins in any one of these pathogens could add further insight as to how bacteria modulate their host cells and cause aberrant transcriptional reprogramming.

## Conclusion

We used a combination of bioinformatic screens and iTRAQ *in vitro* identification of potential nuclear-translocated proteins to stratify and rapidly identify candidate nucleomodulins in *A. phagocytophilum*, an approach easily applied to other intracellular pathogens. By combining data gathered from bioinformatics prediction tools and iTRAQ, 50 *A. phagocytophilum* proteins were identified as potential nucleomodulins. Of the 50, we confirmed that six proteins were capable of localizing to the nucleus on their own, including APH_0455 that is also a T4SS substrate. The identification of novel nuclear translocated proteins provides additional support for the concept of nucleomodulin-mediated reprogramming of cellular functions that improve microbial fitness by promoting extended intracellular survival and more opportunities for transmission.

### Conflict of interest statement

The authors declare that the research was conducted in the absence of any commercial or financial relationships that could be construed as a potential conflict of interest.
