# Supplementary material for: Bioinformatic and mass spectrometry identification of Anaplasma phagocytophilum proteins translocated into host cell nuclei
Source: Front Microbiol. 2015 Feb 6;6:55. doi: 10.3389/fmicb.2015.00055 (PMC4319465; doi:10.3389/fmicb.2015.00055)
Supplement: Supplementary file 1 [file Table1.DOCX]

| **Supplemental Table 1. Primers used for amplification and cloning of potential *A. phagocytophilum* nuclear-translocated protein-encoding genes.** | | |
| --- | --- | --- |
| oligo name | orientation | sequence (5' to 3') |
| APH0062 | forward | GAAGAAAGATCTCGAGCTATGCCTAGGGCGGTTGCCGTG |
| APH0062 | reverse | GAAGCTTGAGCTCGAGTCACACCTTCTGGCGCTGGAGG |
| APH0097 | forward | GAAGAAAGATCTCGAGCTATGCAGCCAAAAATTAGAGTAAGA |
| APH0097 | reverse | GAAGCTTGAGCTCGAGTTTATTTCTCACCATCCGCATTC |
| APH0106 | forward | GAAGAAAGATCTCGAGCTATGTTCAACGGCATAGTTACTC |
| APH0106 | reverse | GAAGCTTGAGCTCGAGTCTATAGGCCATATGTGCGATG |
| APH0135 | forward | GAAGAAAGATCTCGAGCTATGTCTCAGTTAGTGGATGGGAAGGCG |
| APH0135 | reverse | GAAGCTTGAGCTCGAGTTACTCCGAAAGCACCTCTAGGTTCACAGC |
| APH0154 | forward | GAAGAAAGATCTCGAGCTATGGTTGGGTACATTGGGGA |
| APH0154 | reverse | GAAGCTTGAGCTCGAGTCTAGCAAGCCAAAGTATCGTAC |
| APH0240 | forward | GAAGAAAGATCTCGAGCTATGTCAAATACGGTAGTCACG |
| APH0240 | reverse | GAAGCTTGAGCTCGAGTTTAAAATCCGCCCATACCAC |
| APH0280 | forward | GAAGAAAGATCTCGAGCTATGTGGATGAAAAAGGTTGGTA |
| APH0280 | reverse | GAAGCTTGAGCTCGAGTTCAACCAGCCATGCTCTC |
| APH0288 | forward | ATGAGTACAGTCGCTGAAATGGAAGAAAGATCTCGAGCT |
| APH0288 | reverse | GAAGCTTGAGCTCGAGTTTAGACATTCTCTCCCCTTATCTT |
| APH0292 | forward | GAAGAAAGATCTCGAGCTATGTTGGGGAGTTTATCTAAGGA |
| APH0292 | reverse | GAAGCTTGAGCTCGAGTCTAATTCATAAAAGGAAATCCCAACT |
| APH0303 | forward | GAAGAAAGATCTCGAGCTATGGCTGACCATTGGAACAA |
| APH0303 | reverse | GAAGCTTGAGCTCGAGTTTAATCTTCACTATACTGCTTACTCAA |
| APH0339 | forward | GAAGAAAGATCTCGAGCTATGAAGCAATATAAGTTATTGGAGCAA |
| APH0339 | reverse | GAAGCTTGAGCTCGAGTTCACATGCTTAGATATCTATTTACCAG |
| APH0382 | forward | GAAGAAAGATCTCGAGCTATGCATACGCCGCATATATTC |
| APH0382 | reverse | GAAGCTTGAGCTCGAGTCTATGCGCTTCGCGCT |
| APH0385 | forward | GAAGAAAGATCTCGAGCTATGCATACGCCTCGTATATTC |
| APH0385 | reverse | GAAGCTTGAGCTCGAGTTTATGCACTTCGTGATCGTTT |
| APH0397 | forward | ATGGGGAGTATACCAGAGTTTAGGAAGAAAGATCTCGAGCT |
| APH0397 | reverse | GAAGCTTGAGCTCGAGTCTATTCTTGCACAACGGCAT |
| APH0398 | forward | GAAGAAAGATCTCGAGCTATGAAAATTGATGTTGAGGTTATCAA |
| APH0398 | reverse | GAAGCTTGAGCTCGAGTCTAAGCAATTGCGAATAACCTATAC |
| APH0445 | forward | GAAGAAAGATCTCGAGCTATGGTGAATTTGTATAATTTTGATAATCTTG |
| APH0445 | reverse | GAAGCTTGAGCTCGAGTCTAAACCCACCCAACTCGTT |
| APH0455 | forward | GAAGAAAGATCTCGAGCTATGCATATGCCTCGTATATTCAC |
| APH0455 | reverse | GAAGCTTGAGCTCGAGTTCATGCTCTTCGCGCCT |
| APH0469 | forward | GAAGAAAGATCTCGAGCTATGGAAAACTCCCTACTGAAGAT |
| APH0469 | reverse | GAAGCTTGAGCTCGAGTTCAACTTTTCTTCAAAAGAGTAGCT |
| APH0485 | forward | GAAGAAAGATCTCGAGCTATGGAAAAAACTGCTATAGGTGAT |
| APH0485 | reverse | GAAGCTTGAGCTCGAGTTTATATGAGATTACAGCTTTCGGTG |
| APH0515 | forward | GAAGAAAGATCTCGAGCTATGAGAGAAAAACGTCGTCGT |
| APH0515 | reverse | GAAGCTTGAGCTCGAGTTTACTTGCTATCTTTAGAGATAACCTT |
| APH0576 | forward | GAAGAAAGATCTCGAGCTATGCGGGATTCATTGATGGAT |
| APH0576 | reverse | GAAGCTTGAGCTCGAGTTTAGAAAAACCCACGCAATTTC |
| APH0629 | forward | GAAGAAAGATCTCGAGCTATGCGGTCTCATCGTTCA |
| APH0629 | reverse | GAAGCTTGAGCTCGAGTCTACTTTTGCTCAGAAATAATCTTCA |
| APH0659 | forward | GAAGAAAGATCTCGAGCTATGAGACGCCTTATGAATTTGGT |
| APH0659 | reverse | GAAGCTTGAGCTCGAGTTTAGCTCAGACTATCTATATGAGATTG |
| APH0740 | forward | GAAGAAAGATCTCGAGCTATGTTGACAGAAGAAGAAAAGAAAAA |
| APH0740 | reverse | GAAGCTTGAGCTCGAGTCTACCTACCGCGACCTC |
| APH0784 | forward | GAAGAAAGATCTCGAGCTATGAGCAAGGAAATCATAGTAAGG |
| APH0784 | reverse | GAAGCTTGAGCTCGAGTCTACACATTTACCGTATCTAACAAC |
| APH0805 | forward | GAAGAAAGATCTCGAGCTATGAGGAAGTGGGTTATAAACTTTT |
| APH0805 | reverse | GAAGCTTGAGCTCGAGTTCAGGCAATACTCTTTGAAGTG |
| APH0820 | forward | GAAGAAAGATCTCGAGCTATGCTTACACCTAGGAATCTTAGT |
| APH0820 | reverse | GAAGCTTGAGCTCGAGTTTAATGTAATCTGCGTCGTTTGG |
| APH0847 | forward | GAAGAAAGATCTCGAGCTATGCATAATCATGGAAATCCATTAGGT |
| APH0847 | reverse | GAAGCTTGAGCTCGAGTTTATTGTAAATTTGGGAATAACGTACTG |
| APH0906 | forward | GAAGAAAGATCTCGAGCTATGACTCTGCTGCTTAAGCAA |
| APH0906 | reverse | GAAGCTTGAGCTCGAGTTTAATGCTGCTGCTGTGATAC |
| APH0968 | forward | GAAGAAAGATCTCGAGCTATGGAAGAGGGTAAAGTTGTGTT |
| APH0968 | reverse | GAAGCTTGAGCTCGAGTTTAATTCAGAAAAGAACAATCACCAA |
| APH0971 | forward | GAAGAAAGATCTCGAGCTATGCAGCAGTTCTATTGTGTC |
| APH0971 | reverse | GAAGCTTGAGCTCGAGTCTACACACTCTCAAAAAGCGAT |
| APH1023 | forward | GAAGAAAGATCTCGAGCTATGAAGACGTTGGATTTGTATGG |
| APH1023 | reverse | GAAGCTTGAGCTCGAGTCTAGTAATCCACAACGGAGC |
| APH1025 | forward | GAAGAAAGATCTCGAGCTATGAGTAGTGTTGATTTAGATGATTTG |
| APH1025 | reverse | GAAGCTTGAGCTCGAGTTTACTTCAAACTCACCTGAGC |
| APH1027 | forward | GAAGAAAGATCTCGAGCTATGTTGGATAAGAATATTGTCTATACTCCA |
| APH1027 | reverse | GAAGCTTGAGCTCGAGTTCAACCCAATCCAGTTATTCTAAA |
| APH1029 | forward | GAAGAAAGATCTCGAGCTATGAAGCATGATGGTATTGGTC |
| APH1029 | reverse | GAAGCTTGAGCTCGAGTTTATTCCCCTACCTTCTGTATACTAC |
| APH1032 | forward | GAAGAAAGATCTCGAGCTATGACAGAAGGAAGGAAGCC |
| APH1032 | reverse | GAAGCTTGAGCTCGAGTCTACTCCAAAATCTCAGTAATAATACCT |
| APH1097 | forward | GAAGAAAGATCTCGAGCTATGAGCGGTGAGATAAGTAGAG |
| APH1097 | reverse | GAAGCTTGAGCTCGAGTCTATATTCTCATAGGCATAACGATGT |
| APH1098 | forward | GAAGAAAGATCTCGAGCTATGGCTGTATTCGTGCATC |
| APH1098 | reverse | GAAGCTTGAGCTCGAGTCTATGTCTGATGACTGAATATATCTACA |
| APH1099 | forward | GAAGAAAGATCTCGAGCTATGCGCATACTGTTAATAGAAGAT |
| APH1099 | reverse | GAAGCTTGAGCTCGAGTCTAGGCTTCTTCCGCATAATC |
| APH1100 | forward | GAAGAAAGATCTCGAGCTATGGAAGAACACGGTGGTT |
| APH1100 | reverse | GAAGCTTGAGCTCGAGTCTACCGTAGCGCCCTTG |
| APH1151 | forward | GAAGAAAGATCTCGAGCTATGATTCTGTTTTTTGTTTCGTTAGCA |
| APH1151 | reverse | GAAGCTTGAGCTCGAGTCTATCTCCAGTTAACACTCTCAC |
| APH1198 | forward | GAAGAAAGATCTCGAGCTATGGGTGATGCTGTAGAAGTT |
| APH1198 | reverse | GAAGCTTGAGCTCGAGTCTAAATTCCTAGAGCCAATCTGTT |
| APH1239 | forward | GAAGAAAGATCTCGAGCTATGAGATCTAGAAGTAAGCTATTTTTAGGA |
| APH1239 | reverse | GAAGCTTGAGCTCGAGTTTAAAGATGGTTTGTGTAATGAATTCCA |
| APH1263 | forward | GAAGAAAGATCTCGAGCTATGGATAAAAAAGGTCCTAGAATAAACGA |
| APH1263 | reverse | GAAGCTTGAGCTCGAGTTTAAGACCCACCACCAGTTT |
| APH1349 | forward | GAAGAAAGATCTCGAGCTATGATTAGAGTGGGAATAAATGGC |
| APH1349 | reverse | GAAGCTTGAGCTCGAGTTTATAAAAATTTCTGCGCTATTAGGG |
