# Supplementary material for: Bioinformatic and mass spectrometry identification of Anaplasma phagocytophilum proteins translocated into host cell nuclei
Source: Front Microbiol. 2015 Feb 6;6:55. doi: 10.3389/fmicb.2015.00055 (PMC4319465; doi:10.3389/fmicb.2015.00055)
Supplement: Supplementary file 2 [file Table2.DOCX]

| **Supplemental Table 2. Bacterial strains and genomes selected for bioinformatic prediction of nuclear localized proteins.** | | | | | | | |
| --- | --- | --- | --- | --- | --- | --- | --- |
| **Organism/strain** | **Group** | **Gram Staining** | **Size Mb** | **GC %** | **chr*** | **plasmids**** | **GenBank** |
| *Anaplasma phagocytophilum* HZ | Alphaproteobacteria | - | 1.47 | 42 | 1 |  | CP000235 |
| *Brucella abortus* biovar 1 str. 9-941 | Alphaproteobacteria | - | 3.29 | 57 | 2 |  | AE017223 |
| *Chlamydia trachomatis* D/UW-3/CX | Chlamydiales | - | 1.05 | 41 | 1 | 1 | CP000051 |
| *Chlamydophila pneumoniae* AR39 | Chlamydiales | - | 1.23 | 40 | 1 |  | AE001273 |
| *Coxiella burnetii* RSA 493 | Gammaproteobacteria | - | 2.03 | 43 | 1 | 1 | AE016828 |
| *Ehrlichia chaffeensis* str. Arkansas | Alphaproteobacteria | - | 1.18 | 30 | 1 |  | CP000236 |
| *Francisella tularensis* subsp. holarctica | Gammaproteobacteria | - | 1.90 | 32 | 1 |  | AM233362 |
| *Legionella pneumophila* subsp. | Gammaproteobacteria | - | 3.40 | 38 | 1 |  | AE017354 |
| *Listeria monocytogenes* str. 4b F2365 | Firmicutes | + | 2.91 | 37 | 1 |  | AE017262 |
| *Mycobacterium tuberculosis* CDC1551 | Actinobacteria | + | 4.40 | 66 | 1 |  | AE000516 |
| *Rickettsia prowazekii* str. Madrid E | Alphaproteobacteria | - | 1.11 | 29 | 1 |  | AJ235269 |
| *Yersinia pestis* CO92 | Gammaproteobacteria | - | 4.83 | 48 | 1 | 3 | AL590842 |
| *number of chromosomes; ** number of plasmids | | | | | | | |
