# Supplementary material for: Bioinformatic and mass spectrometry identification of Anaplasma phagocytophilum proteins translocated into host cell nuclei
Source: Front Microbiol. 2015 Feb 6;6:55. doi: 10.3389/fmicb.2015.00055 (PMC4319465; doi:10.3389/fmicb.2015.00055)
Supplement: Supplementary file 3 [file Table3.DOCX]

| **Supplemental Table 3. Prediction of bacterial proteins for nuclear localization and the presence of an nuclear localization sequence (NLS).** | | | | | |
| --- | --- | --- | --- | --- | --- |
| **Organism/strain** | **DNA** | **RefSeq** | **ORFs** | **Nuclear ProtComp** | **NLS MultiLoc** |
| *Anaplasma phagocytophilum* HZ | Chromosome | NC_007797 | 1264 | 120 | 4 |
| *Brucella abortus* biovar 1 str. 9-941 | Chromosome I | NC_006932 | 2030 | 99 | 5 |
|  | Chromosome II | NC_006933 | 1055 | 47 | 6 |
| *Chlamydia trachomatis* D/UW-3/CX | Chromosome | NC_000117 | 895 | 58 | 7 |
|  | Plasmid pCTA | NC_007430 | 8 | 1 | 0 |
| *Chlamydophila pneumoniae* AR39 | Chromosome | NC_002179 | 1112 | 74 | 10 |
| *Coxiella burnetii* RSA 493 | Chromosome | NC_002971 | 2016 | 174 | 29 |
|  | Plasmid pQpH1 | NC_004704 | 36 | 5 | 0 |
| *Ehrlichia chaffeensis* str. Arkansas | Chromosome | NC_007799 | 1105 | 141 | 12 |
| *Francisella tularensis* subsp. holarctica | Chromosome | NC_007880 | 1754 | 92 | 9 |
| *Legionella pneumophila* subsp. pneumophila | Chromosome | NC_002942 | 2942 | 163 | 29 |
| *Listeria monocytogenes* str. 4b F2365 | Chromosome | NC_002973 | 2821 | 97 | 16 |
| *Mycobacterium tuberculosis* CDC1551 | Chromosome | NC_002755 | 4189 | 247 | 14 |
| *Rickettsia prowazekii* str. Madrid E | Chromosome | NC_000963 | 835 | 70 | 11 |
| *Yersinia pestis* CO92 | Chromosome | NC_003143 | 3885 | 173 | 6 |
|  | Plasmid pCD1 | NC_003131 | 72 | 7 | 0 |
|  | Plasmid pPCP1 | NC_003132 | 9 | 2 | 0 |
|  | Plasmid pMT1 | NC_003134 | 101 | 9 | 0 |
| **TOTALS** |  |  | **26129** | **1579** | **158** |
