# Supplementary material for: Bioinformatic and mass spectrometry identification of Anaplasma phagocytophilum proteins translocated into host cell nuclei
Source: Front Microbiol. 2015 Feb 6;6:55. doi: 10.3389/fmicb.2015.00055 (PMC4319465; doi:10.3389/fmicb.2015.00055)
Supplement: Supplementary file 4 [file DataSheet1.DOCX]

**Supplemental Figure 1.** Algorithm used to discern potential nuclear translocated proteins in *A. phagocytophilum* HZ genome and among 11 additional genomes of intracellular bacteria. See methods for detailed description of programs and algorithm decision points.

**Supplemental Figure 2.** The six *A. phagocytophilum* candidate genes found to localize to the nucleus of HEK-293T cells were also transfected in PLB 985 promyelocytic leukemia cells after candidate genes were fused to GFP. 24 h post-transfection, cells were stained with DAPI and imaged. All 6 candidates also localized to the nucleus of PLB 985 granulocytes, reproducing similar morphologic distributions as with APH_0455.
