# Supplementary material for: Bioinformatic and mass spectrometry identification of Anaplasma phagocytophilum proteins translocated into host cell nuclei
Source: Front Microbiol. 2015 Feb 6;6:55. doi: 10.3389/fmicb.2015.00055 (PMC4319465; doi:10.3389/fmicb.2015.00055)
Supplement: Supplementary file 5 [file DataSheet2.DOCX]

**Supplemental methods**

***Selection of intracellular pathogens with genomes available for bioinformatic analysis of predicted nuclear localized proteins.***

Among the main intracellular bacterial pathogens for which genomic sequences have been determined, species were selected that represent phylogenetically diverse groups, including both Gram negative and Gram positive bacteria. These bacteria are also known to affect host cell gene transcription and function. Interestingly, most of the selected bacteria are able to propagate within human defense cells, including granulocytes, monocytes or macrophages, and contain type IV secretion systems that are known to facilitate translocation of bacterial molecules from the bacterial cytoplasm into the host cell. Twelve bacterial species were selected based on their relevance as human and animal pathogens (Supplementary Table 1). All selected bacteria had to fulfill the following criteria: 1) have an intracellular life cycle stage; 2) have a genome sequence available; 3) are known to effect host cell gene transcription, based on available global transcriptional analysis data.

**Comparative genomics analysis of candidate ORFs.**

Conceivably, proteins that encoded by orthologs of genes present in the genomes of extracellular bacteria are potentially not required as fitness factors for intracellular survival. To focus study only on potentially high-reward candidates, we used OrthoMCL DB was used to identify orthologs of candidate genes in extracellular bacterial genomes (Chen et al., 2006). OrthoMCL is a genome-scale algorithm for ortholog protein sequences which identifies ORFs shared by two or more species/genomes. OrthoMCL starts by looking for reciprocal best hits within each genome as putative ortholog pairs. In a preliminary analysis, a cutoff E value < 10^-100^ was used to identify orthologs of the 184 candidate ORFs in extracellular bacteria genomes, using the *E. coli* genome as a reference. Of these 184 ORFs, 26 were discarded based on the degree of conservation among both intracellular and extracellular bacteria. Supplemental Table 3 shows a summary of the results by bacterial species of the successive steps of this preliminary search.

**References**

Chen, F., et al., OrthoMCL-DB: querying a comprehensive multi-species collection of ortholog groups. Nucleic Acids Res, 2006. 34 (Database issue): p. D363-8
